# Preparation Process Optimization of Glycolipids from *Dendrobium officinale* and the Difference in Antioxidant Effects Compared with Ascorbic Acid

**DOI:** 10.3390/nu16213664

**Published:** 2024-10-28

**Authors:** Yan Long, Jiajing Yang, Hongfei Ji, Xiao Han, Yuting Fan, Keyao Dai, Haiyu Ji, Juan Yu

**Affiliations:** 1 Yantai Key Laboratory of Characteristic Agricultural Bioresource Conservation & Germplasm Innovative Utilization, School of Life Sciences, Yantai University, Yantai 264005, China; 18282780162@163.com (Y.L.); yangfan2812@163.com (J.Y.); hongfei10052024@163.com (H.J.); hanxiao7721@163.com (X.H.); fyting7766@163.com (Y.F.); haiyu11456@163.com (H.J.); 2 College of Food Science and Engineering, Tianjin University of Science and Technology, Tianjin 300457, China; dai13389086120@163.com

**Keywords:** *Dendrobium officinale* glycolipids, structural analysis, antioxidant effects, intestinal flora metabolism

## Abstract

Background**:** *Dendrobium officinale* glycolipids (DOG), often left as residues after hot water extraction for polysaccharide production, are often discarded. Methods: This study investigates the optimal extraction of DOG using response surface methodology, focusing on liquid–solid ratios, ethanol concentrations, extraction temperatures, and extraction times, while preliminarily analyzing DOG’s structural properties. Additionally, the differences in antioxidant effects between DOG and ascorbic acid based on intestinal flora metabolism were further evaluated. Results: The optimal parameters for DOG extraction were determined as follows: liquid–solid ratio of 20 mL/g, ethanol concentration of 70%, extraction temperature of 70 °C, and extraction time of 2.5 h, yielding 2.64 ± 0.18%. In addition, DOG was identified as a diglyceride, mainly composed of glucose, mannose, linoleic acid, 9,12,15-octadecatrienoic acid, and presented certain direct free radicals scavenging effects. In animal experiments, unlike the direct free scavenging effects of ascorbic acid, DOG increased intestinal *Bacteroides acidifaciens* abundance in mice, up-regulated piceatannol expression, and down-regulated 1-naphthol expression, which contributed to antioxidant effects by enhancing the activities of SOD and GSH-Px while reducing MDA content. Conclusions: DOG was a diglyceride isolated from *D. officinale* residues after hot water extraction, and presented strong antioxidant effects by regulating intestinal flora metabolism. These findings could promote the efficient utilization of *D. officinale* and support further development of DOG in functional food applications.

## 1. Introduction

*Dendrobium officinale* is a perennial herb from the *Dendrobium* genus in the *Orchidaceae* family, known for its medicinal and food properties [1,2]. Recent studies have revealed that *D. officinale* contains various bioactive components like polysaccharides and alkaloids, which present potential effects on enhancing immunity, regulating blood glucose levels, as well as providing antioxidant and antiaging benefits [3,4,5]. Typically, these active components are prepared using hot water extraction followed by ethanol precipitation or column chromatography separation [6,7]. However, the residues left after water extraction are often discarded without being utilized effectively, resulting in waste of resources. Glycolipids, complex compounds composed of lipids and sugars that are insoluble in water [8], can be categorized into glyceroglycolipids and sphingoglycolipids. Glyceroglycolipids employ glycerol as the backbone, with fatty acids and sugars linked by glycosidic bonds, and are widely found in animals, plants, and microorganisms. Glycolipids play crucial roles in maintaining normal physiological cell function while also contributing to immune defense mechanisms and disease development [9]. This study aims to prepare glycolipids from the water-extracted residues of *D. officinale* and to evaluate their antioxidant activity in vitro and in vivo, facilitating a comprehensive approach to the development and utilization of *D. officinale*.

During normal physiological processes, the human body generates reactive oxygen species (ROS) through metabolic activities, which are crucial for certain cellular functions (such as signaling pathways and immune responses). However, excessive ROS can lead to health problems [10,11,12]. Under normal circumstances, the body’s antioxidant defense system (including superoxide dismutase, catalase, and glutathione peroxidase, etc.) can effectively neutralize excessive ROS [13]. However, when there is an imbalance between the production of ROS and their clearance through antioxidase, these accumulated substances can damage various cellular components, such as proteins, lipids, and DNA, disrupting normal cellular structure and function, thereby contributing to a range of health issues, including inflammatory bowel disease and diabetic ulcers [14,15]. Therefore, effective management of oxidative damage plays a crucial role in maintaining optimal health and preventing/treating diverse ailments.

Gut microbiota refers to the microbial community inhabiting the human gastrointestinal tract and coexisting with the host organism. This community participates in food digestion and absorption and plays pivotal roles in immune regulation, metabolic control, psychological behavior modulation, and other essential functions [16,17]. Gut microbiota can ferment indigestible cellulose and polysaccharides present in food into easily absorbed beneficial metabolites such as short-chain fatty acids, which can provide energy sources for cellular metabolism and serve important functions like regulating immune response, promoting normal colonic mucosal barrier function, and inhibiting proliferation of pathogenic microecology [18,19]. Moreover, certain gut microbiota can produce antioxidant substances, such as superoxide dismutase and glutathione peroxidase, to mitigate oxidative stress damage, while an imbalance in gut microbiota may lead to increased levels of oxidative stress [20]. Therefore, research on the relationship between intestinal flora and antioxidant mechanisms can provide more effective means and guidance for improving overall health.

In this study, *D. officinale* glycolipid (DOG) was prepared using hot-water-extracted residues as raw materials, and four factors—liquid–solid ratios, ethanol concentrations, extraction temperatures, and extraction times—were optimized using response surface methodology. Following this, the structure of DOG was preliminarily analyzed. Additionally, an oxidative damage animal model was established to investigate the antioxidant activities of DOG and ascorbic acid based on intestinal flora metabolism. These findings will provide valuable data to support further development of DOG in the functional foods field, and promote the efficient utilization of *D. officinale* raw materials.

## 2. Materials and Methods

### 2.1. Materials

The dried stems of *D. officinale* were bought from Yunnan Shuairun Technology Co., Ltd. (Kunming, China). Anhydrous ethanol was brought from Jinan Yande Biotechnology Co., Ltd. (Jinan, China). Antioxidant capacity assay kit (DPPH, ABTS method), superoxide dismutase (SOD) assay kit, glutathione peroxidase (GSH-PX) assay kit, and malondialdehyde (MDA) assay kit were purchased from Nanjing Jiancheng Bioengineering Institute (Nanjing, China).

### 2.2. Preparation of DOG

The dried *D. officinale* was crushed to uniform powder (300 mesh) with a Chigo pulverizer (Jinhua, China), and then immersed in deionized water under 80 °C for 4 h, the extraction was repeated 3 times, and all leaching solution were mixed for polysaccharides preparation. The residues obtained after water extraction were subjected to drying and utilized as the raw materials in this paper. The raw materials were subjected to ethanol soaking and heating for enhanced dissolution efficiency. Following repeated extraction 2 times, the supernatant was combined, concentrated via a vacuum rotary evaporation, and freeze-dried to obtain DOG. The yields were determined by the ratios of DOG weights to that of dried residues. The response surface experiment was designed by adjusting the liquid–solid ratios, ethanol concentrations, extraction temperatures, and extraction time to optimize the extraction process of DOG, with the yields serving as the sole indicator [21]. The experimental design and results are presented in Table 1.

### 2.3. UV Full Wavelength Scanning and Characteristic Functional Groups Determination of DOG

The UV scanning spectrum of DOG ranging from 200 nm to 800 nm was determined through a microplate reader (Bio-Tek, Charlotte, VT, USA). In addition, Fourier Transformation Infrared Spectroscopy (FTIR) was employed for analyzing characteristic functional groups of DOG. Briefly, 140 mg KBr and 1.0 mg DOG powder ad were weighed, crushed into a pellet, then the spectrum at 4000–400 cm^−1^ with 4 cm^−1^ resolution and 16 scan repetitions was determined and analyzed through a FTIR spectrometer (Bruker VECTOR-22, Karlsruhe, Germany) [22].

### 2.4. Monomer Compositions Determination of DOG

Gas chromatography–mass spectrometry (GC-MS) was used to detect the monomeric composition of DOG. The 5 mg DOG was weighed, subjected to complete hydrolysis using 2 mol/L trifluoroacetic acid of 2 mL under 110 °C for 4 h, acetylated with acetic anhydride under 90 °C for 0.5 h. After dried with N_2_ under 70 °C, the sample was extracted using 1 mL dichloromethane, then directly determined and analyzed by a triple quadrupole GC-MS (7000C/US1521U204, Agilent, CA, USA). The analysis was performed using the following parameters: loading amount of 2 μL, chromatographic column of HP-5ms (30 m × 250 μm × 0.25 μm), temperature program started at 100 °C for 1 min, then ramped up to 200 °C at a rate of 50 °C/min for 2 min, followed by an increase to 250 °C at a rate of 5 °C/min for 3 min. Finally, the temperature was raised to 280 °C at a rate of 30 °C/min for 6 min. The injection port temperature was set at 250 °C and helium gas served as the carrier gas. The spectral database NIST14.L was utilized for compound identification [23].

### 2.5. Antioxidant Activity Assay In Vitro

The scavenging capabilities of DOG on DPPH and ABTS free radicals were assessed following the provided guidelines. To investigate its antioxidant properties, DOG was tested at five different concentrations (0.25, 0.50, 1.00, 2.00, and 4.00 mg/mL), while ascorbic acid was used as a positive control in equivalent amounts [24].

### 2.6. Animal Experimental Design

A total of 50 male Kunming mice, aged 6 weeks (25 ± 2 g), were procured from Jinan Pengyue Experimental Animal Breeding Co. LTD. The mice were accommodated in a relative humidity ranging between 45%~55%, while the temperature was maintained at 20~25 °C. Following an acclimatization period, all mice were randomly divided into five groups (10 mice per group): blank group, model group, ascorbic acid group, low-dose DOG treatment group (DOG-L, 50 mg/kg), and high-dose DOG treatment group (DOG-H, 100 mg/kg).

The establishment of oxidative damage mice model was built upon prior research studies with some modifications [25]. The blank and model groups were orally administered 0.2 mL of normal saline (0.9%), while the ascorbic acid group received a dosage of 50 mg/kg of ascorbic acid for 21 days. Meanwhile, the DOG-L and DOG-H groups were given DOG at corresponding dosages respectively. On day 8, all experimental groups except the blank group received cyclophosphamide (CTX) injections at a dosage of 60 mg/kg for 3 days. On day 22, the mice sera and feces were collected for further determination.

### 2.7. Antioxidant Activity Assay In Vivo

The activities of antioxidant enzymes including SOD and GSH-Px in mice sera, and the MDA levels were determined utilizing the kits provided and following the instructions specific to each kit. Besides, the antioxidant levels of mice sera in each group were also analyzed [26].

### 2.8. 16S rRNA Amplicon Detection Method

Intestinal flora diversity analysis of mice was conducted using 16S rRNA sequencing. On the 22nd day, samples of feces were collected from the blank, model, ascorbic acid and DOG-H groups for detecting genomic DNA by agarose gel electrophoresis (concentration of 1%). Following that, specific primers containing barcodes were synthesized in order to target the V3 and V4 regions for amplification through PCR. The resulting products underwent purification using agarose gel electrophoresis and quantification utilizing the QuantiFluor™-ST Blue Fluorescence Quantitation System. Subsequently, selected fragments with labels were subjected to PCR amplification while generating single-stranded DNA fragments via NaOH treatment. These fragments were then identified using illumina sequencing technology. Finally, the qualitative and quantitative analysis of intestinal flora was performed by comparing data of Sequence Read Archive database (http://www.ncbi.nlm.nih.gov/Traces/sra, accessed on 20 February 2024) [27].

### 2.9. Non-Target Metabolites Detection Method

Untargeted metabolomics was employed to analyze the intestinal metabolites in mice, which facilitated the identification of various small molecule metabolites present in the samples [28]. On day 22, fecal samples from model, ascorbic acid and DOG-H groups were collected and processed into powder under liquid nitrogen conditions. Subsequently, 100 mg samples were weighed and mixed with methanol solution (80%). After 5 min rest under ice bath, the supernatants were obtained through centrifugation (15,000× *g*, 20 min, 4 °C) for subsequent analysis using liquid chromatography-mass spectrometry (LC-MS), which utilized Dionex Ultimate 3000 system equipped with Thermo Syncronis C18 column (2.1 mm × 100 mm). The identification process was performed using Trace Finder software (version 3.2.0). Gradient elution conditions: 0~1 min, 95% A; 1~5 min, 95%~40% A; 5~8 min, 40%~0% A; 8~11 min, 0% A; 11~14 min, 0%~40% A; 14~15 min, 40%~95% A; 15~18 min, 95% A. A represented water containing 0.1% formic acid and 2 mmoL/L ammonium formate, while B represented acetonitrile.

### 2.10. Statistical Analysis

The data analysis was conducted using SPSS software (version 24.0), and the results were presented as mean ± SD with a minimum of three replicates. Statistical significance was determined through one-way variance analysis, considering *p* < 0.05 and *p* < 0.01.

## 3. Results and Discussions

### 3.1. Effects of Extraction Parameters on DOG Yields

The response surface method was employed to optimize the parameters for DOG extraction, including liquid–solid ratios, ethanol concentrations, extraction temperatures, and extraction time. The design scheme and determination results were presented in Table 1. The findings demonstrated a close agreement between the measured and predicted values, indicating a high level of reliability in the experimental results.

Design-Expert software (Version: 13.0.5.0 64-bit) was applied to evaluate the ANOVA for DOG extraction results [29], and the quadratic model could be presented with the following equation in terms of coded factors:

DOG yields = 2.64 + 0.36*A* + 0.04*B* + 0.03*C* + 0.06*D* + 0.01*AB* − 0.06*AC* + 0.04*AD* − 0.05*BC* + 0.07*BD* + 0.05*CD* − 0.57*A*^2^ − 0.33*B*^2^ − 0.21*C*^2^ − 0.12*D*^2^

Moreover, the variance analysis for quadratic model is displayed in Table 2. 

As presented, the results revealed that the coefficients *A*, *D*, *BD*, *A*^2^, *B*^2^, *C*^2^, and *D*^2^ had a high level of significance (*p* < 0.01), while *B*, *C*, *AC*, and *BC* also presented obvious effects on DOG yields (*p* < 0.05). However, the interaction effects of *AB* and *AD* did not show any significant difference in DOG yields. Additionally, the *p* < 0.0001 of model and *p* = 0.7258 of lack-of-fit value indicated that deviations showed insignificant effects and support the validity of fitted model. Moreover, the difference between the adjusted R^2^ value (0.9874) and predicted R^2^ value (0.9770) was 0.0104, suggesting a strong correlation between predicted and actual DOG extraction yields, consistent with Table 1 [30].

### 3.2. Effects of Parameters Interaction on DOG Yields

**Figure 1:**
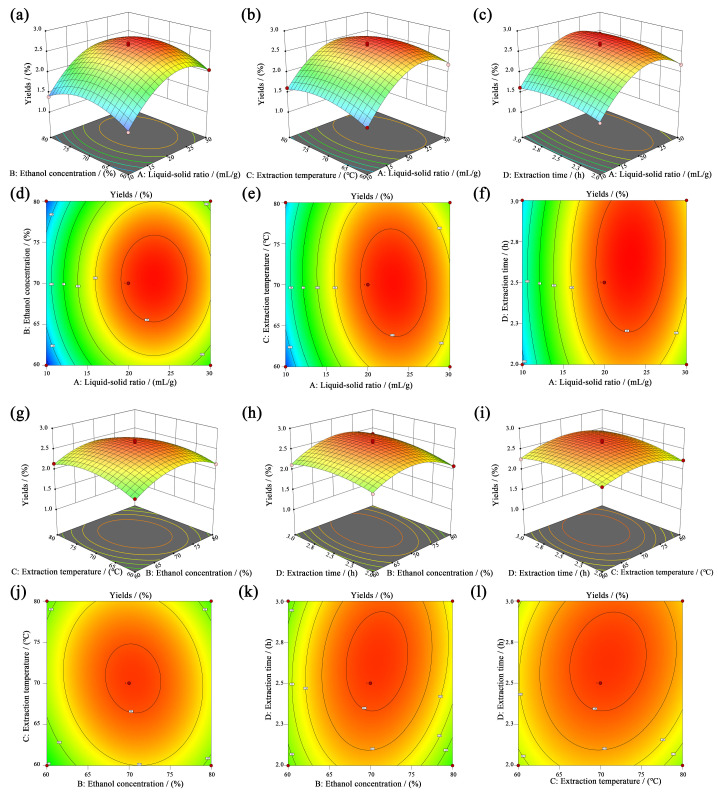
Response surface (**a**–**c**,**g**–**i**) and contour plots (**d**–**f**,**j**–**l**) of the DOG extraction with various variables interaction including liquid–solid ratios, ethanol concentrations, extraction temperatures, extraction time. The colors transition from blue to red indicated the increasing DOG yields.

Figure 1 illustrates the interaction impacts of varying liquid–solid ratios (A, 10~30 mL/g), ethanol concentrations (B, 60%~80%), extraction temperatures (C, 60~80 °C), and extraction times (D, 2.0~3.0 h) on DOG yields. All response surface curves exhibited peak values within the specified experimental ranges, indicating a reasonable range of factors. The contour plots in elliptical or circular shapes indicated significant or indistinctive interaction effects among these variables, the color gradient in the figures could be used to represent the magnitude of response values, with a transition from blue (indicating low values) to red (indicating high values), and darker colors indicating proximity to extreme values. Consequently, it can be observed that the combination of *AC* (b, e), *BC* (g, j), *BD* (h, k), *CD* (i, l) has pronounced interaction effects (*p* < 0.05) on DOG extraction yields, which aligns with previous findings [31].

### 3.3. Validation Experiment for DOG Extraction

The software analysis results indicated that the optimal approach for DOG extraction was determined as follows: liquid–solid ratio of 23.2104 mL/g, ethanol concentration of 70.8566%, extraction temperature of 70.4833 °C, Extraction time of 2.6619 h, and the predicted yields of 2.7058%. Considering the feasibility and convenience of industrial production [32], the optimal process parameters were adjusted as liquid–solid ratio of 20 mL/g, ethanol concentration of 70%, extraction temperature of 70 °C, and extraction time of 2.5 h. These parameters were subjected to validation experiments, resulting in DOG yields of 2.64 ± 0.18%.

### 3.4. Major Functional Groups Analysis of DOG

**Figure 2:**
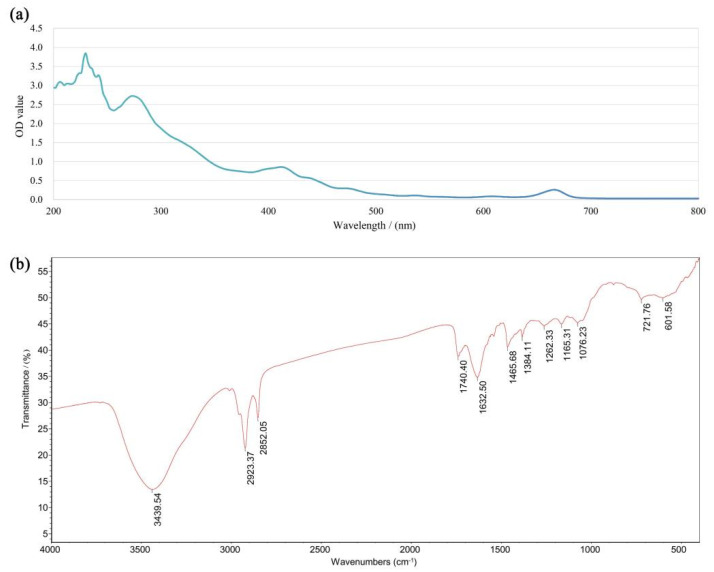
UV full wavelength scanning (**a**) and FTIR (**b**) spectra of DOG.

The UV full wavelength scanning (a) and FTIR (b) spectra of DOG are presented in Figure 2. The absorption of saturated fatty acids is relatively weaker, with their absorption peaks typically falling within the lower wavelength range (200–210 nm). In contrast, unsaturated fatty acids exhibit a stronger capacity for ultraviolet absorption, with their absorption peaks located in the higher wavelength range (220–230 nm). The results in Figure 2a suggest that DOG contain both saturated and unsaturated fatty acids. In addition, the absorptions at around 280 nm and 410 nm indicate the potential presence of double bonds and conjugation in DOG [33,34].

Figure 2b displays the FTIR spectrum of DOG. As shown, the huge absorption peaks at 3439.54 cm^−1^ in DOG are attributed to O–H stretching, indicating some hydrophilic capacity. The signals at 2923.37 cm^−1^ and 2852.05 cm^−1^ suggest the presence of CH_2_ and CH, respectively [35]. The absorptions at 1740.40 cm^−1^ and 1632.50 cm^−1^ can be assigned to the stretching vibrations of the carbonyl group (C=O), indicating the presence of esters [36]. The bands at 1465.68 cm^−1^ and 1384.11 cm^−1^ can be attributed to the antisymmetric and symmetric bending vibrations of CH_3_. The characteristic absorption peak at 1262.33 cm^−1^ primarily corresponds to the vibrational mode of the hydroxyl group in the primary alcohol, while the bands at 1165.31 cm^−1^ and 1076.23 cm^−1^ may be attributed to C-O-C and C-O-H vibrations [37,38]. Therefore, DOG exhibits the characteristic functional groups of glycolipids.

### 3.5. Monomer Compositions Analysis of DOG

The monomer compositions of DOG were determined by GC-MS and the results are presented in Table 3.

As displayed, the glycerol 1,2-diacetate constitutes 20.43% of DOG, indicating that DOG primarily consists of diglycerides. The presence of D-Mannitol, hexaacetate (7.42%), and D-Glucitol, hexaacetate (62.36%) suggests that the glycosyl moiety in DOG mainly comprises glucose and mannose. Furthermore, the identification of these particular fatty acids including hexadecanoic acid (1.71%), linoleic acid (4.38%), 9,12,15-octadecatrienoic acid (2.78%), octadecanoic acid (0.92) in DOG was supported by the detection of relevant ethyl esters. The structural framework of glycolipids is characterized by a glycerol backbone covalently linked to monosaccharide residues and fatty acids through O-glycosidic bonds, which exhibit widespread distribution across plant, animal, and bacterial kingdoms. These glycolipids are involved in cell-to-cell communication, immune response modulation, photosynthetic electron transport [39,40]. Therefore, the antioxidant effects of DOG on oxidative damage in mice were further evaluated.

### 3.6. Antioxidant Activity In Vitro of DOG

**Figure 3:**
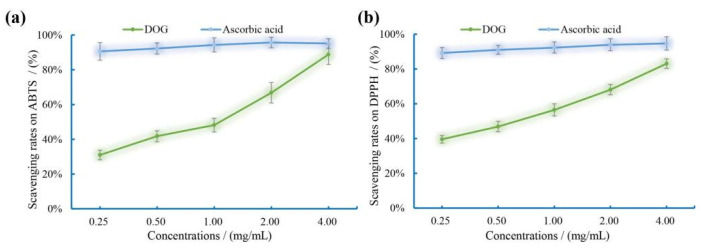
Scavenging rates of DOG and ascorbic acid on ABTS (**a**) and DPPH (**b**) free radicals.

The scavenging rates of DOG on ABTS and DPPH free radicals were determined with ascorbic acid as positive control, and the results are shown in Figure 3. As presented, with the increasing concentrations of DOG and ascorbic acid from 0.25 to 4.00 mg/mL, the scavenging effects of both compounds on ABTS and DPPH free radicals exhibited a similar trend. Ascorbic acid demonstrated potent antioxidant activity in vitro, even at the lowest concentration (0.25 mg/mL), achieving approximately 90% scavenging capacity on these free radicals, while further increases in concentrations did not yield significant improvements in antioxidant activity. Meanwhile, with increasing DOG concentrations, the average ABTS free radical scavenging rates increased from 30.97% to 88.78%, and the average DPPH free radical scavenging rate increased from 39.56% to 83.09%, indicating that DOG exhibited certain capacities to directly scavenge free radicals [41].

### 3.7. Antioxidant Activity In Vivo of DOG on Oxidative Damaged Mice

**Figure 4:**
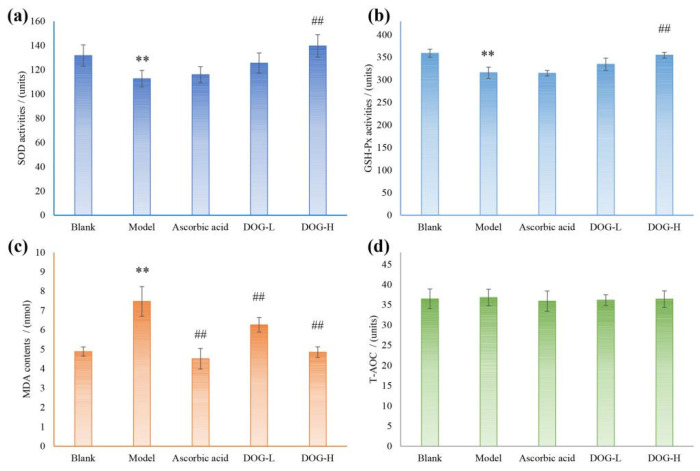
Antioxidant effects of DOG on oxidative damaged mice sera. (**a**), SOD activities; (**b**), GSH-Px activities; (**c**), MDA contents; (**d**), total antioxidant capacity. Note: **, *p* < 0.01 compared with blank group; ^##^, *p* < 0.01 compared with model group.

The antioxidant indicators in mice sera of each group were determined using relevant kits, and the results are presented in Figure 4. As depicted in Figure 4a,b, compared with blank group, the activities of SOD and GSH-Px in mice sera of model group both exhibited a significant decrease (*p* < 0.01), indicating that intraperitoneal injection of cyclophosphamide severely impacted the body’s antioxidant system. No substantial improvement was observed after intervention with ascorbic acid, suggesting the inability to enhance antioxidant enzyme activities within the body. Conversely, following DOG gavage interventions, varying degrees of improvement were observed in these two antioxidant enzymes’ activities within mice sera, indicating that DOG could enhance overall antioxidant levels by increasing antioxidant enzyme activities. As shown in Figure 4c, the MDA contents in mice sera were remarkably increased (*p* < 0.01) of model group compared with that of blank group, suggesting that the antioxidant system was suppressed by cyclophosphamide. After being treated by ascorbic acid and DOG, the MDA contents were significantly reduced compared with model group, suggesting antioxidant effects in vivo, while the action mechanisms may be different. DOG exerts antioxidant functions in vivo by enhancing the activities of associated enzymes, whereas ascorbic acid acts as an antioxidant by directly scavenging free radicals [42]. In addition, the direct scavenging capacities of mice sera in each group on free radicals did not exhibit any significant differences, indicating the robust self-coordination ability within the body’s peripheral blood.

### 3.8. Intestinal Microbial Diversity Results of Oxidative Damaged Mice

**Figure 5:**
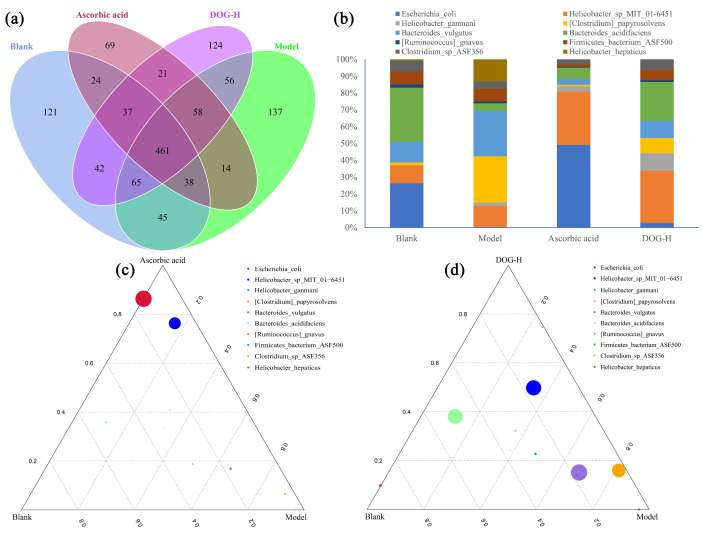
Effects of ascorbic acid and DOG on intestinal microbial diversity. (**a**), Venn diagram; (**b**), relative contents at species level; (**c**), ternary plot among blank, model and ascorbic acid groups; (**d**), ternary plot among blank, model and DOG groups.

The effects of ascorbic acid and DOG on intestinal microbial diversity of cyclophosphamide-induced oxidative damaged mice were determined, as shown in Figure 5. In Figure 5a, the Venn diagram demonstrates that there were 461 commonly owned Operational Taxonomic Units (OTUs) among these groups, while the specific OTUs in blank, model, ascorbic acid and DOG groups were 121, 137, 69, 124, respectively, suggesting the obvious impacts of various treatments on the intestinal microbial diversity. Figure 5b shows the relative contents of intestinal microorganisms at species level. Compared with blank group, the contents of *Clostridium papyrosolvens*, *Bacteroides vulgatus*, and *Helicobacter hepaticus* in model group were obviously increased, while the contents of *Escherichia coli* and *Bacteroides acidifaciens* were remarkably descended, indicating that the inflammatory response, oxidative damage and immunosuppression were induced by cyclophosphamide in mice of model group, which has been substantiated across various species, including humans [43,44,45]. Compared with the model group, the contents of *Escherichia coli* and *Helicobacter* sp. *MIT 01-6451* in ascorbic acid group were significantly increased while the contents of *Clostridium papyrosolvens* and *Bacteroides vulgatus* were reduced. Meanwhile, DOG-H group presented higher contents of *Helicobacter* sp. *MIT 01-6451* and *Bacteroides acidifaciens*, and lower proportions of *Clostridium papyrosolvens* and *Bacteroides vulgatus*. These results suggested that the antioxidant effects of DOG and ascorbic acid were different. As shown in Figure 5c,d, it could be observed more intuitively that *Clostridium papyrosolvens* and *Bacteroides vulgatus* in model group mainly played roles in inhibiting the antioxidant system of the body, while *Helicobacter* sp*. MIT 01-6451* in ascorbic acid and DOG groups might reflect the antioxidant and immunoregulatory levels in vivo [46]. *Escherichia coli* mainly played important roles as an indicator in oxidative damage mice after ascorbic acid treatment, while *Bacteroides acidifaciens* could improve the DOG-induced antioxidant activity via protecting liver metabolism [47].

### 3.9. Differential Metabolites Analysis of Between Model and Ascorbic Acid Groups

The non-targeted metabolomics technique was applied for the quantitative and qualitative analysis of intestinal metabolites in mice of each group. Table 4 demonstrates the intestinal metabolites with significant expression differences between the ascorbic acid and model groups. The results show the expression of 25 metabolites with significant differences, with 15 being downregulated and 10 being upregulated.

**Figure 6:**
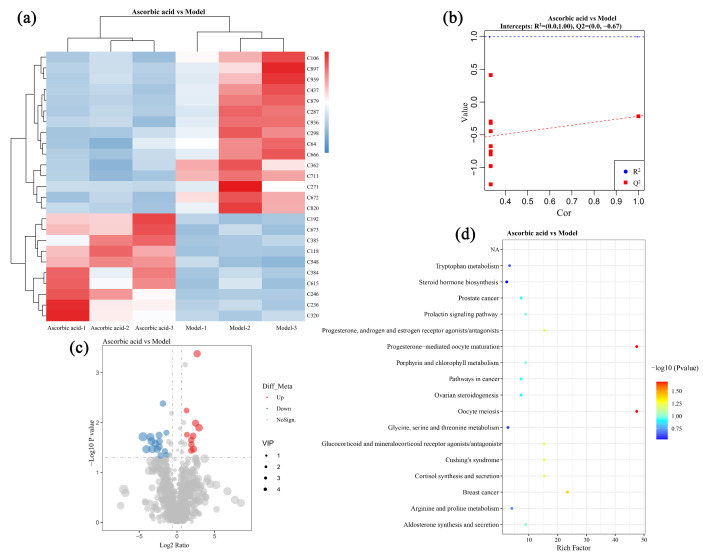
Differential metabolites analysis in ascorbic acid group compared with model group. (**a**), Heatmap with metabolites ID; (**b**), model overfitting analysis; (**c**), volcano map; (**d**), KEGG enrichment plot.

The findings from further analysis of these 25 differential metabolites are presented in Figure 6. In Figure 6a, a color gradient from blue to red signifies an increase in metabolite concentration, thereby facilitating a clearer visual representation of the expression differences among various metabolites. The results of the Partial Least Squares Discriminant Analysis depicted in Figure 6b indicated that R^2^ exceeded Q^2^ and approached 1, with the Q^2^ regression line exhibiting a negative intercept on the Y-axis, suggesting that the model fitted well without overfitting. The volcano plot illustrated in Figure 6c reveals that the red points corresponding to metabolites were significantly upregulated in ascorbic acid group compared with the model group, while blue points denote those that were downregulated, and gray points represent metabolites with no significant expression changes. These results demonstrate that ascorbic acid intervention led to a greater number of downregulated metabolites compared to upregulated ones. Furthermore, signal pathway enrichment analysis for these metabolites shown in Figure 6d identified two significantly altered pathways: Progesterone (C897)-mediated oocyte meiosis and maturation. Studies have shown that progesterone can promote the expressions of antioxidant enzymes such as superoxide dismutase (SOD) and glutathione peroxidase (GPX) in cells, especially in oocytes, and improve their antioxidant activity through relevant signaling pathways [48]. However, the results in this study showed that ascorbic acid intervention led to a significant downregulation of progesterone levels in oxidatively stressed mice, indicating that the signaling pathways mediated by progesterone were also inhibited. These findings are consistent with those of previous studies reporting that ascorbic acid failed to enhance the activities of antioxidant enzymes.

### 3.10. Differential Metabolites Analysis of Between Model and DOG Groups

Table 5 presents the intestinal metabolites exhibiting notable variations in expressions between the DOG-H and model group. The findings indicated that a total of 63 metabolites displayed significant differences, with 35 showing downregulation and 28 displaying upregulation.

**Figure 7:**
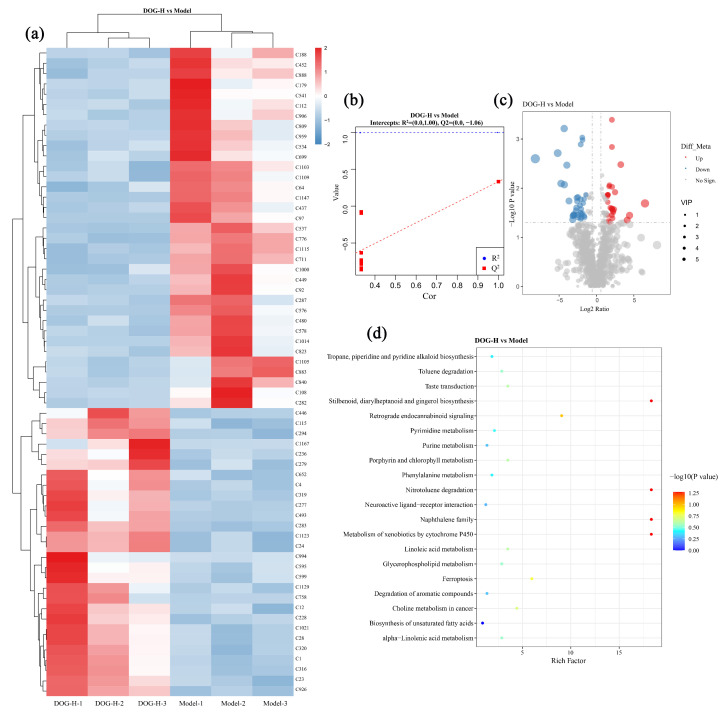
Differential metabolites analysis in DOG-H group compared with model group: (**a**) Heatmap with metabolites ID; (**b**) model overfitting analysis; (**c**) volcano map, and (**d**) KEGG enrichment plot.

Figure 7a–c provides a more intuitive perspective on the regulatory effects of DOG on oxidative damaged mice, revealing a greater diversity of differential metabolites compared with ascorbic acid and indicating high level of data reliability. In Figure 7d, the signal pathways are enriched for all these metabolites, and four pathways are identified: 4-methylphenol (C228)-mediated nitrotoluene degradation, piceatannol (C1167)-mediated stilbenoid, diarylheptanoid and gingerol biosynthesis, and 1-naphthol (C97)-mediated metabolism of xenobiotics by cytochrome P450 and naphthalene family. In this paper, DOG primarily functioned as an antioxidant by enhancing the activities of relevant enzymes in the body, thereby diminishing the significance of 4-methylphenol’s potential direct free radical scavenging effects. Studies have demonstrated that piceatannol cannot be synthesized by certain microorganisms during fermentation and may enhance the activities of antioxidant enzymes in the body [49]. Conversely, 1-naphthol and the metabolites could induce a substantial production of ROS within organisms, disrupting the inherent REDOX balance and triggering oxidative stress [50]. Consequently, the antioxidant mechanism of DOG was closely associated with up-regulating piceatannol expression while down-regulating 1-naphthol expression, finally leading to an increase in antioxidant enzyme activities and protecting effects against oxidative damage induced by cyclophosphamide.

## 4. Conclusions

In conclusion, the optimal extraction parameters for DOG, determined via response surface methodology, were a liquid–solid ratio of 20 mL/g, ethanol concentration of 70%, extraction temperature of 70 °C, and extraction time of 2.5 h, yielding 2.64 ± 0.18%. DOG was identified as a diglyceride mainly composed of glucose, mannose, linoleic acid, and 9,12,15-octadecatrienoic acid, exhibiting notable free radical scavenging effects. In animal experiments, unlike the direct free radical scavenging action of ascorbic acid, DOG primarily enhanced the abundance of *Bacteroides acidifaciens* in the gastrointestinal tract, up-regulated piceatannol expression, and down-regulated 1-naphthol expression, thereby increasing SOD and GSH-Px activities and reducing MDA levels in mice sera. However, further investigations are still required to gain more comprehensive insights into the specific glycolipid components involved, and additional research is required to verify the metabolic pathways and functions of the characteristic differential metabolites due to limitations in mass spectrometry analysis.

## Figures and Tables

**Table 1:** Response surface test design and results.

Run	A: Liquid–Solid Ratios	B: Ethanol Concentrations	C: Extraction Temperatures	D: Extraction Time	Predicted Yields	Actual Yields
	(mL/g)	(%)	(°C)	(h)	(%)	(%)
1	30	70	70	3.0	2.39	2.43
2	10	60	70	2.5	1.35	1.32
3	10	70	60	2.5	1.41	1.43
4	20	70	80	2.0	2.21	2.23
5	30	70	60	2.5	2.23	2.19
6	20	70	80	3.0	2.43	2.41
7	20	70	60	3.0	2.28	2.26
8	30	70	70	2.0	2.19	2.19
9	10	70	70	2.0	1.55	1.53
10	30	70	80	2.5	2.18	2.15
11	20	80	70	3.0	2.34	2.35
12	20	70	70	2.5	2.64	2.56
13	10	70	80	2.5	1.58	1.61
14	10	70	70	3.0	1.61	1.62
15	20	80	80	2.5	2.10	2.08
16	20	80	70	2.0	2.08	2.09
17	20	60	60	2.5	1.98	2.01
18	20	60	80	2.5	2.13	2.15
19	20	70	70	2.5	2.64	2.63
20	30	60	70	2.5	2.05	2.06
21	20	80	60	2.5	2.15	2.14
22	20	70	70	2.5	2.64	2.67
23	20	60	70	2.0	2.14	2.12
24	20	70	60	2.0	2.25	2.27
25	20	70	70	2.5	2.64	2.61
26	30	80	70	2.5	2.13	2.16
27	10	80	70	2.5	1.40	1.39
28	20	60	70	3.0	2.14	2.12
29	20	70	70	2.5	2.64	2.71

**Table 2:** Variance analysis for quadratic model.

Source	Sum of Squares	DF	Mean Square	F-Value	*p*-Value	Significance
Model	4.14	14	0.2960	158.01	<0.0001	**
*A*-Liquid–solid ratio	1.53	1	1.53	814.82	<0.0001	**
*B*-Ethanol concentration	0.0154	1	0.0154	8.22	0.0124	*
*C*-Extraction temperature	0.0091	1	0.0091	4.84	0.0450	*
*D*-Extraction time	0.0481	1	0.0481	25.69	0.0002	**
*AB*	0.0002	1	0.0002	0.1201	0.7341	
*AC*	0.0121	1	0.0121	6.46	0.0235	*
*AD*	0.0056	1	0.0056	3.00	0.1051	
*BC*	0.0100	1	0.0100	5.34	0.0366	*
*BD*	0.0169	1	0.0169	9.02	0.0095	**
*CD*	0.0090	1	0.0090	4.82	0.0455	*
*A* ^2^	2.11	1	2.11	1128.53	<0.0001	**
*B* ^2^	0.7157	1	0.7157	382.01	<0.0001	**
*C* ^2^	0.2989	1	0.2989	159.55	<0.0001	**
*D* ^2^	0.1070	1	0.1070	57.10	<0.0001	**
Residual	0.0262	14	0.0019			
Lack of Fit	0.0131	10	0.0013	0.3996	0.8908	not significant
Pure Error	0.0131	4	0.0033			
Cor Total	4.17	28				
Adjusted R^2^	0.9874		R^2^	0.9937		
Predicted R^2^	0.9770		C.V.	2.04		

Note: *, *p* < 0.05; **, *p* < 0.01.

**Table 3:** Monomer compositions of DOG.

No.	Compound Descriptions	Molecular Formula	Retention Time (min)	Molecular Mass (Da)	Proportions (%)
1	Glycerol 1,2-diacetate	C_7_H_12_O_5_	4.79	176.07	20.43%
2	Hexadecanoic acid, ethyl ester	C_18_H_36_O_2_	10.55	284.27	1.71%
3	D-Mannitol, hexaacetate	C_18_H_26_O_12_	12.45	434.14	7.42%
4	D-Glucitol, hexaacetate	C_18_H_26_O_12_	12.66	434.14	62.36%
5	Linoleic acid ethyl ester	C_20_H_36_O_2_	13.02	308.27	4.38%
6	9,12,15-Octadecatrienoic acid, ethyl ester, (Z,Z,Z)-	C_20_H_34_O_2_	13.13	306.26	2.78%
7	Octadecanoic acid, ethyl ester	C_20_H_40_O_2_	13.42	312.30	0.92%

**Table 4:** Differential metabolites in ascorbic acid group compared with model group.

Metabolites ID	Compound Descriptions	Molecular Formula	Retention Time (min)	*p* Values	Up.Down
C879	PG(18:3(9Z,12Z,15Z)/18:3(6Z,9Z,12Z))	C_42_H_71_O_10_P	4.67	0.0192	down
C271	8-Isoprostaglandin F2a	C_20_H_34_O_5_	5.55	0.0341	down
C287	ACar 15:1	C_22_H_42_NO_4_	8.19	0.0194	down
C897	Progesterone	C_21_H_30_O_2_	6.76	0.0235	down
C959	Stercobilin	C_33_H_46_N_4_O_6_	10.18	0.0338	down
C298	ACar 20:2	C_27_ H_50_NO_4_	9.36	0.0262	down
C820	PC (22:6e/18:5)	C_48_H_76_NO_7_P	7.68	0.0343	down
C711	N-lactoyl-phenylalanine	C_12_H_15_NO_4_	1.17	0.0179	down
C106	2-(1H-1,2,3-benzotriazol-1-yl)-N-(2,3-dihydro-1H-inden-2-yl)acetamide	C_17_H_16_ N_4_O	13.14	0.0305	down
C437	DL-o-Tyrosine	C_9_H_11_NO_3_	2.87	0.0239	down
C936	SM(d18:0/16:0)	C_39_H_81_N_2_O_6_P	4.67	0.0459	down
C672	N,N-Dimethylsphing-4-enine	C_20_H_41_NO_2_	9.15	0.0041	down
C666	N-(3-Oxohexanoyl)homoserine lactone	C_10_H_15_NO_4_	9.18	0.0372	down
C362	Ceramide (d18:1/16:0)	C_34_H_67_NO_3_	10.06	0.0159	down
C64	11-Oxoetiocholanolone	C_19_H_28_O_3_	13.87	0.0444	down
C673	N-[(4-hydroxy-3-methoxyphenyl)methyl]-8-methylnonanamide	C_18_H_29_NO_3_	13.72	0.0057	up
C548	L-2-Amino-3-oxobutanoic acid	C_4_H_7_NO_3_	0.56	0.0174	up
C236	5,7-dimethyl-2-phenylpyrazolo[1,5-a]pyrimidine	C_14_H_13_N_3_	13.24	0.0223	up
C320	All-Trans-13,14-Dihydroretinol	C_20_H_32_O	5.11	0.0362	up
C384	Creatinine	C_4_H_7_N_3_O	0.57	0.0274	up
C246	5-Methoxyindoleacetic acid	C_11_H_11_NO_3_	4.06	0.0185	up
C615	LysoPC(14:0)	C_22_H_46_NO_7_P	6.51	0.0340	up
C385	Cryptotanshinone	C_19_H_20_O_3_	4.3	0.0102	up
C118	2,4-Dihydroxybenzoic acid	C_7_H_6_O_4_	0.83	0.0004	up
C192	3-Methoxycinnamic acid	C_10_H_10_O_3_	4.27	0.0126	up

**Table 5:** Differential metabolites in DOG-H group compared with model group.

Metabolites ID	Compound Descriptions	Molecular Formula	Retention Time (min)	*p* Values	Up.Down
C1014	(2,3,9,17,22R)-2,3,14,20,22-Pentahydroxyergost-7-en-6-one	C_28_H_46_O_6_	6.28	0.0025	down
C576	LPC 14:0	C_22_H_46_NO_7_P	8.60	0.0019	down
C97	1-Naphthol	C_10_H_8_O	13.00	0.0080	down
C537	Inosine	C_10_H_12_N_4_O_5_	4.05	0.0006	down
C108	2-(1H-indol-3-yl)acetic acid	C_10_H_9_NO_2_	15.19	0.0085	down
C92	1-Methyladenosine	C_11_H_15_N_5_O_4_	0.55	0.0034	down
C959	Stercobilin	C_33_H_46_N_4_O_6_	10.18	0.0182	down
C480	FRH	C_21_H_30_N_8_O_4_	10.88	0.0435	down
C179	3-Hydroxy-5, 8-tetradecadiencarnitine	C_19_H_25_BN_4_O_4_	6.17	0.0366	down
C809	PC (19:0/19:1)	C_46_H_90_NO_8_P	10.45	0.0359	down
C840	PC(18:1(11Z)/20:0)	C_46_H_90_NO_8_P	13.30	0.0341	down
C287	ACar 15:1	C_22_H_42_NO_4_	8.19	0.0350	down
C1105	(13E,16E,19E)-Docosatri-13,16,19-enoic acid	C_22_H_38_O_2_	9.62	0.0286	down
C188	3-Indoleacrylic acid	C_11_H_9_NO_2_	3.22	0.0251	down
C437	DL-o-Tyrosine	C_9_H_11_NO_3_	2.87	0.0157	down
C282	ACar 12:1	C_19_H_36_NO_4_	5.93	0.0184	down
C883	Phosphocholine	C_5_H_14_NO_4_P	13.19	0.0348	down
C1147	PC (18:2e/20:4)	C_46_H_82_NO_7_P	13.17	0.0152	down
C823	PC (9:0/9:0)	C_26_H_52_NO_8_P	8.43	0.0390	down
C906	Protectin D1	C_22_H_32_O_4_	6.73	0.0224	down
C699	N-Arachidonoyl-L-serine	C_23_H_37_NO_4_	13.02	0.0420	down
C112	2-(3,5-dimethyl-1H-pyrazol-4-yl)-5-methoxybenzoic acid	C_13_H_14_N_2_O_3_	4.05	0.0314	down
C578	LPC 15:0	C_23_H_48_NO_7_P	6.87	0.0168	down
C711	N-lactoyl-phenylalanine	C_12_H_15_NO_4_	1.17	0.0013	down
C1103	(+/−)11(12)-EET	C_20_H_32_O_3_	7.44	0.0266	down
C1000	Uridine	C_9_H_12_N_2_O_6_	0.58	0.0339	down
C541	Kahweol	C_20_H_26_O_3_	2.98	0.0397	down
C1115	2-Arachidonoylglycerol	C_23_H_38_O_4_	9.03	0.0009	down
C776	PC (17:1/17:2)	C_42_H_78_NO_8_P	9.77	0.0010	down
C1109	12-HETE	C_20_H_32_O_3_	7.26	0.0328	down
C449	D-Phenylalanine	C_9_H_11_NO_2_	1.43	0.0202	down
C452	Ecgonine	C_9_H_15_NO_3_	3.25	0.0166	down
C64	11-Oxoetiocholanolone	C_19_H_28_O_3_	13.87	0.0366	down
C534	indoline-2-carboxylic acid	C_9_H_9_NO_2_	1.35	0.0391	down
C888	Pimelylcarnitine	C_14_H_25_NO_6_	5.25	0.0136	down
C279	9-Oxo-ODE	C_18_H_30_O_3_	6.44	0.0136	up
C28	(9Z)-(7S,8S)-Dihydroxyoctadecenoic acid	C_18_H_34_O_4_	6.07	0.0136	up
C1021	12(13)-DiHOME	C_18_H_34_O_4_	9.23	0.0147	up
C115	2,3-dihydroxypropyl 12-methyltridecanoate	C_17_H_34_O_4_	6.79	0.0192	up
C446	Dodecanoylcarnitine	C_19_H_37_NO_4_	7.78	0.0353	up
C320	All-Trans-13,14-Dihydroretinol	C_20_H_32_O	5.11	0.0136	up
C23	(6R,7S)-6,7-Epoxyoctadecanoic acid	C_16_H_26_O_5_	7.41	0.0136	up
C595	LPE 18:2	C_23_H_44_NO_7_P	6.93	0.0462	up
C1	(+/−)12(13)-DiHOME	C_18_H_34_O_4_	6.47	0.0092	up
C316	Adrenic acid	C_22_H_36_O_2_	0.59	0.0087	up
C12	(2R)-(9Z,12Z,15Z)-2-Hydroperoxyoctadecatri-9,12,15-enoic acid	C_20_H_40_O_2_	6.03	0.0489	up
C1129	D-(+)-Maltose	C_12_H_22_O_11_	4.32	0.0249	up
C236	5,7-dimethyl-2-phenylpyrazolo[1,5-a]pyrimidine	C_14_H_13_N_3_	13.24	0.0259	up
C283	ACar 12:3	C_19_H_32_NO_4_	5.27	0.0004	up
C277	9-HpODE	C_18_H_32_O_4_	6.5	0.0354	up
C926	SM (d17:0/23:1)	C_45_H_91_N_2_O_6_P	3.38	0.0015	up
C228	4-Methylphenol	C_7_H_8_O	3.69	0.0094	up
C1123	9-HpODE	C_18_H_32_O_4_	6.53	0.0270	up
C24	(7S,8S)-DiHODE	C_18_H_32_O_4_	6.44	0.0261	up
C599	LPE 20:5	C_25_H_42_NO_7_P	6.93	0.0409	up
C652	Matricin	C_17_H_22_O_5_	5.43	0.0290	up
C493	Ginkgoic acid	C_22_H_34_O_3_	8.89	0.0307	up
C294	ACar 18:2	C_25_H_46_NO_4_	7.41	0.0269	up
C4	(11E,15Z)-9,10,13-trihydroxyoctadeca-11,15-dienoic acid	C_18_H_32_O_5_	4.78	0.0121	up
C319	all-cis-4,7,10,13,16-Docosapentaenoic acid	C_22_H_34_O_2_	6.47	0.0033	up
C994	Trolox	C_14_H_18_O_4_	5.78	0.0445	up
C758	PC (16:0/17:0)	C_41_H_82_NO_8_P	8.25	0.0358	up
C1167	Piceatannol	C_14_H_12_O_4_	0.58	0.0204	up

## Data Availability

The raw data supporting the conclusions of this article will be made available by the authors on request.
